# Assessment of the Public Health Threats Posed by Vector-Borne Disease in the United Kingdom (UK)

**DOI:** 10.3390/ijerph15102145

**Published:** 2018-09-29

**Authors:** Jolyon M. Medlock, Kayleigh M. Hansford, Alexander G. C. Vaux, Ben Cull, Emma Gillingham, Steve Leach

**Affiliations:** 1 Medical Entomology Group, Public Health England, Emergency Response Department, Porton Down, Salisbury, Wiltshire SP4 0JG, UK; kayleigh.hansford@phe.gov.uk (K.M.H.); alexander.vaux@phe.gov.uk (A.G.C.V.); ben.cull@phe.gov.uk (B.C.); emma.gillingham@phe.gov.uk (E.G.); steve.leach@phe.gov.uk (S.L.); 2 Health Protection Research Unit in Environmental Change and Health, Porton Down, Salisbury, Wiltshire SP4 0JG, UK; 3 Health Protection Research Unit in Emerging and Zoonotic Infections, Porton Down, Salisbury, Wiltshire SP4 0JG, UK

**Keywords:** vector-borne disease, UK, mosquito, tick, Lyme, arbovirus

## Abstract

In recent years, the known distribution of vector-borne diseases in Europe has changed, with much new information also available now on the status of vectors in the United Kingdom (UK). For example, in 2016, the UK reported their first detection of the non-native mosquito *Aedes albopictus*, which is a known vector for dengue and chikungunya virus. In 2010, *Culex modestus*, a principal mosquito vector for West Nile virus was detected in large numbers in the Thames estuary. For tick-borne diseases, data on the changing distribution of the Lyme borreliosis tick vector, *Ixodes ricinus*, has recently been published, at a time when there has been an increase in the numbers of reported human cases of Lyme disease. This paper brings together the latest surveillance data and pertinent research on vector-borne disease in the UK, and its relevance to public health. It highlights the need for continued vector surveillance systems to monitor our native mosquito and tick fauna, as well as the need to expand surveillance for invasive species. It illustrates the importance of maintaining surveillance capacity that is sufficient to ensure accurate and timely disease risk assessment to help mitigate the UK’s changing emerging infectious disease risks, especially in a time of climatic and environmental change and increasing global connectivity.

## 1. Background

The status of vector-borne disease has changed significantly over the last 15 years in Europe. Europe now has increasingly regular outbreaks or clusters of local cases of West Nile [1], chikungunya [2], and dengue viruses [3], transmitted by mosquitoes that, in the case of the latter two viruses, have only recently expanded to large parts of Europe in the last decade. Local malaria transmission has returned [4], Lyme borreliosis is on the increase [5], and tick-borne encephalitis virus is moving further north in Europe [6]. For veterinary health, Bluetongue virus has appeared in new geographic areas [7], and Schmallenberg virus has emerged [8]. There continue to be the detection of new pathogens, such as *Borrelia miyamotoi* [9] and various tick-borne rickettsiae [10]. This comes at a time of global climate change and large-scale environmental change; both of which drive changes in the habitats of the arthropod vectors and their animal hosts. The global emergency that is associated with Zika virus crystallised the very real risk from emerging vector-borne diseases, and the ease with which they can spread. Europe, including the United Kingdom (UK), is not immune to these disease threats.

As part of UK government preparedness and response to emerging infections, a cross-agency, UK-wide (i.e., including devolved administrations) one-health approach has been developed through the Human Animal Infections Risk Surveillance (HAIRS) group to assess and respond to emerging infectious disease issues. Public Health England (PHE) Medical Entomology group, in partnership with colleagues in the Animal and Plant Health Agency (APHA), conduct passive, active, and enhanced surveillance of disease vectors and conduct research, including with academic partners, to inform the HAIRS group and the Advisory Committee on Dangerous Pathogens (ACDP), which in turn, informs the Chief Medical and Veterinary Officers in the various parts of the UK. This opinion paper brings together surveillance and research data that informs the current public health threats to the UK posed by vector-borne disease. It is not intended to be a review of all topics, but to focus on the primary risks and emerging issues that have arisen or are current, and to highlight areas for further investigation. It is principally focused on infectious diseases that are transmitted by mosquitoes and ticks that concern public health.

## 2. Mosquitoes & Mosquito-Borne Disease

### 2.1. Chikungunya, Dengue and Zika Viruses and Invasive Aedes Mosquitoes

The UK currently has no known established populations of non-native *Aedes* mosquito vector species competent for chikungunya, dengue, or Zika virus. However, the potential for such mosquitoes to establish in the UK, which have proved to be invasive and competent virus vectors elsewhere in Europe, strongly suggest that ongoing UK mosquito surveillance and risk assessments are warranted.

#### 2.1.1. Importance of *Aedes aegypti*

Zika virus (ZIKV), which transmitted by the mosquito *Aedes aegypti* L., caused much global alarm in 2015/2016 when it emerged in South America, leading to millions of human cases right across the region, including in the Caribbean and North America, and it was implicated in Congenital Zika syndrome [11,12]. For the majority, it remained a mild or even subclinical disease, however, its effects on newborns caused grave concern [13], with many European governments questioning the vector status of locally established mosquitoes, and the potential for incursion of invasive mosquito vector species. The spread of invasive *Aedes* mosquitoes in Europe, and incursions into the UK, has been a concern for medical entomologists for over a decade [14,15]; however, the global crisis of ZIKV raised its profile higher up the agenda.

At the time ZIKV was the latest in a list of viruses that are transmitted by *Aedes* subgenus *Stegomyia* mosquitoes that has established throughout the Americas, following yellow fever, dengue, and chikungunya viruses before it [16]. In a similar fashion to chikungunya in 2013/2014, Zika spread rapidly across the Americas and the Caribbean, exploiting the same vector and the same mode of dissemination through human travel. The congenital effects of the virus, however, increased its global profile and the profile of its disease vector. The primary vector, *Ae. aegypti.* is one of the most successful synanthropic colonisers and it is one of the most invasive insect species globally [17]. Its success is testament to its ability to exploit the way in which we store water, the way in which we dispose and store our rubbish, and its ability to benefit from the impacts of extreme weather, such as hurricanes and heavy rain. Its control and eradication is an endless task and the Holy Grail for medical entomologists. This can only be achieved through consistent community effort, which proves a constant challenge wherever the mosquito occurs [18]. It is a vector waiting for a suitable virus and its proficiency has been highlighted in these recent outbreaks. Novel genetic control methods to control *Ae. aegypti* are being developed, and the promotion of integrated approaches to control, with community engagement, will be crucial to successfully manage this mosquito globally [19,20].

#### 2.1.2. *Aedes aegypti* in Europe

In Europe, *Ae. aegypti* was once established across the Mediterranean Basin up until the 1950s, with some records as late as the 1970s, where for the most part it was eradicated, except perhaps for a small population on the Black Sea coast [21]. Prior to its eradication it was implicated in large scale outbreaks of dengue virus (DENV), with more than one million cases in Greece in the 1920s [22]. It first “re-appeared” in Europe in 2005 on Madeira, having never previously been endemic there. Within seven years of the mosquito arriving, and following an imported case and subsequent local transmission, more than 2000 human cases of dengue fever were reported on the island in 2012, which was the first significant outbreak of dengue in Europe for many decades [3]. Since then, concerted surveillance efforts coordinated by VectorNet entomologists as part of a continent-wide effort to map vectors and build local capacity for surveillance, have confirmed an eastern population of the mosquito expanding around the Black Sea in Georgia, Russia and Northern Turkey [23]. It is possible that *Ae. aegypti* will expand into Greece, and also possibly from Madeira into Portugal.

#### 2.1.3. *Aedes aegypti* in the UK—Past, Present, and Future

As for the UK, the species is considered to be climatically limited and remains absent. There have only ever been three published reports of the mosquito in the UK, firstly in 1865 in Swansea (associated with a local outbreak of yellow fever virus [24]), in 1919 in Epping Forest in Essex, and more recently, in 2014 in Merseyside [25]. The source of this recent discovery in north-west England remains a mystery. None of these findings constituted evidence of local established populations, however, and the mosquito remains absent from the UK. It should be noted that due to the medical importance of this species, it is heavily studied within the laboratory setting within UK insectaries, and every effort should continue to be made to ensure that accidental releases never occur.

During the Zika outbreaks, questions were raised over the potential for the survival of *Ae. aegypti* in the UK and their potential for importation through aeroplanes and boats into UK ports. Climatic assessments of *Ae. aegypti* survival in Europe are generally based upon temperature thresholds of a January isotherm of 10 °C, and annual mean temperature of 15 °C. Although fairly crude estimates, they remain a useful guide in risk assessment. By appraising data from the UK Meteorological Office (www.metoffice.gov.uk), in a UK context and for comparison, January isotherms for Scotland are 4–5 °C, in England mostly 5–6 °C, with 7 °C in southwest Cornwall. In some years, it may exceed 8 °C in a few localities; but, all far below the required 10 °C threshold. Similarly, annual mean temperatures are also some way below the required 15 °C mean threshold. They vary across the UK from 4 to 11 °C, and even in a warm summer, such as 2015, mean temperatures were only 0.5–1 °C higher than this 4–11 °C range. Of course these are crude climatic estimates, and further modelling is required to more accurately assess future risk in a changing climate.

There remains a theoretical risk that the mosquito could survive in colder temperatures in sheltered environments indoors or underground, but this has never been reported as an issue in Europe [25]. *Aedes aegypti* does not undergo a winter diapause, and therefore, needs to remain active all year, with a general seasonal peak of activity being recorded in Madeira from April/May through to October [3]. There is also a theoretical risk that an imported mosquito could survive during a warm summer only to die out at the onset of winter. Indeed, this occurred in 1865 in Swansea [21]. However, the reports of *Ae. aegypti* arriving into Europe on aeroplanes are limited [26], and despite surveillance at 37 UK sea and airports since 2016, there has been no detection so far of *Ae. aegypti* into the UK [27]. That the mosquito has not been detected yet in the UK does not mean that surveillance should not continue, but rather it should be ongoing, with any European spread of this mosquito routinely monitored.

It seems unlikely that this species will establish in the UK over the next few decades unless there is some behavioural adaptation of the mosquito to more temperate climes. Therefore, viruses or strains of viruses that are only transmitted by *Aedes aegypti* are unlikely to currently be a concern for endemic transmission in the UK, notwithstanding their importance for returning travellers that were infected abroad. The role of native UK and other invasive mosquitoes as potential vectors of medically and veterinary important viruses remains an open question, and continues to be the subject of laboratory research. The demonstration of viral competence in a laboratory setting at defined temperatures, following the mosquito feeding on infected blood, is a useful laboratory test. However it is not necessarily indicative of vector status in the wild, although it can sometimes be interpreted as such. Competence in the field is dependent upon a number of factors, including biting rate, host preferences (including the dominance of non-human blood hosts), the diurnal cycle of temperature, and the impact that this has on the extrinsic incubation of the virus in the mosquito, as well as the number of infected humans.

#### 2.1.4. *Aedes albopictus* in Europe

The Asian tiger mosquito, *Aedes albopictus* (Skuse), on the other hand, has been suggested for some time as a species of more concern for the UK [14]. The first European report of *Ae. albopictus* came from Albania in 1979, followed by Italy in 1990, having been imported as drought-resistant eggs on used tyres (a global commodity for re-treading and recycling) from the United States (US) into Genoa [28]. The mosquito was then detected in 15 other localities, and since then the mosquito has managed to colonise almost all parts of Italy, becoming a persistent nuisance species [29].

Although being unable to disperse very far itself, it has managed to disseminate and establish widely in Europe though movement in vehicles. It has now been reported in 28 European countries, and it is highly abundant in southern Europe, including Italy, Spain, southern France, the Balkans and Greece. Last year it was reported in Gibraltar and Portugal [30], and it shows an ever increasing spread into northern France and Germany [31]. Its vector role was long suspected and feared in Europe [32], and indeed an adaptation of chikungunya virus (CHIKV) to the mosquito in La Reunion led to more than 250,000 local cases on the island, more than one million cases of viral disease in India, and subsequently a local outbreak of more than 200 cases in northern Italy [33,34]. Since then, there have been isolated cases of CHIKV and DENV associated with *Ae. albopictus* in France and Croatia in 2010, local cases of DENV and CHIKV in France in 2014, and an isolated case of CHIKV in Spain in 2015 [28,35]. Further cases of DENV and CHIKV were reported in France in 2015 and 2017 respectively [36]. Italy also reported a second large outbreak of CHIKV in 2017, with more than 350 cases [2].

#### 2.1.5. *Aedes albopictus*—Risks for the UK

A number of models developed over the last 15 years suggest that the UK climate is warm enough for the survival of *Ae. albopictus*, with a number of months of activity predicted [14,37,38]. When set in a European context, with the current UK climatic factors compared to those in much of the rest of Europe, they are likely to be less abundant [38], although coupled with the increasing number of infected travellers returning with these viruses, suggests that local transmission of an arbovirus is theoretically possible [39]. The extrinsic incubation of these viruses in the mosquito is temperature dependent, and further modelling is needed to ascertain whether during a hot summer these viruses could develop in *Ae. albopictus* in the UK, something that certainly appears to have occurred in France for both DENV and CHIKV.

**Figure 1:**
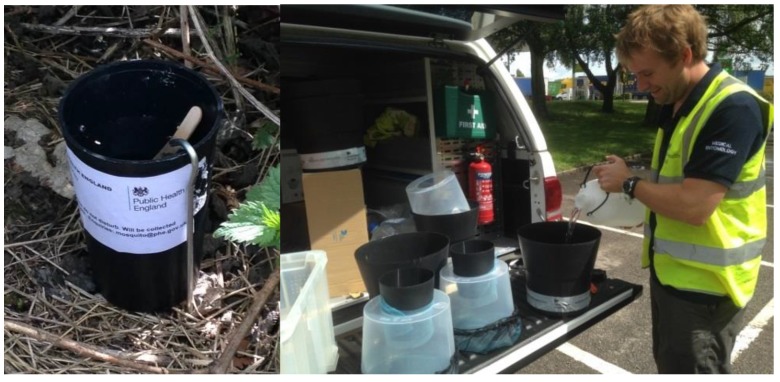
Invasive mosquito surveillance using ovitraps and Gravid *Aedes* traps at Ports of entry and transport hubs.

**Figure 2:**
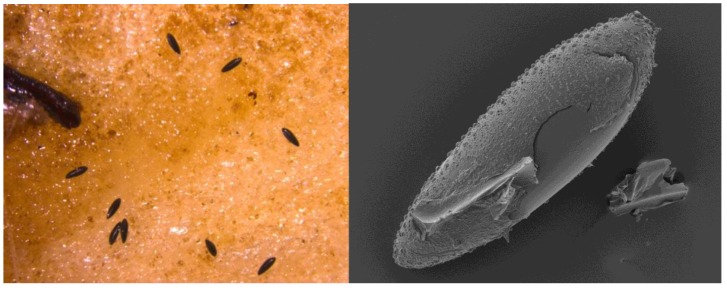
Detection of *Aedes albopictus* eggs in 2016 in Kent: On substrate and under Scanning Electron Microscope (SEM).

Surveillance for *Ae. albopictus* in the UK was initiated by the Health Protection Agency (HPA) and colleagues in 2010 at a number of ports [40,41] and now there are more than 37 UK ports sustaining surveillance for invasive mosquitoes in England, Wales, Scotland, and Northern Ireland [27]. So far there is no evidence of either the detection or establishment of this mosquito at UK ports. In 2014, this programme of surveillance was extended to the highway systems (Figure 1), on account of the dissemination of the mosquito through France in vehicles [41]. A number of motorway service stations and truck stops have been monitored in south-east England since 2014, and 37 eggs of *Ae. albopictus* were detected at a truck stop near Folkestone in 2016 (Figure 2) [42]. Local control efforts coordinated by the local authority with support from PHE were instigated and no further mosquitoes were found.

A second interception was detected at a truck stop near Ashford in 2017 [27], thus highlighting that these incursions would continue. Progress has been made with regards to preparing for future incursions: including providing training in mosquito surveillance to local authorities; preparing contingency plans for dealing with incursions and dealing with any possible establishment of the mosquito; as well as management around any local human cases of arboviral disease that may occur.

The challenge is intercepting these mosquitoes and controlling them, or minimising their chances to establish. This remains a huge task, requiring a robust and satisfactorily resourced active, targeted monitoring, as well as promotion of existing passive surveillance, such as that managed by PHE and partners. Invasive mosquitoes can best be controlled, and their establishment prevented, if there is surveillance at the most likely routes of importation. Stemming the tide for now is important to minimise the immediate risk. It is likely that these mosquitoes may establish in the UK in the future, and may result in nuisance and vector-related concerns. Keeping the UK free of these disease vectors is important, but having plans to deal with local arboviral disease outbreaks, such as has occurred in France, is also important for any future eventualities. It is important that we prepare for a future in which UK public health may have to deal with clusters of human cases that are attributed to viruses that we only recently considered to be tropical ones. The mosquito and the viruses are exploiting our globalisation of commodities and the increase in air travel. Imported mosquitoes and returned infected travellers are the key ingredients for local transmission and a warming climate will only tend to increase the associated risk over time.

### 2.2. West Nile Virus and Culex Mosquitoes

In contrast to the *Aedes*-transmitted arboviruses, West Nile virus (WNV) is primarily a zoonotic *Culex* mosquito-borne flavivirus cycling in birds capable of both horizontal and vertical transmission. Humans and horses exhibit clinical disease, but they essentially act as dead end hosts. Global concerns over the public health importance of this virus heightened following its appearance in the United States (US) in 1999. Since then, it subsequently spread rapidly throughout most US states in 10 years, leading to more than 40,000 human cases, with more than 1600 deaths [43]. Primarily transmitted by *Culex* mosquitoes, interest in the possible circulation of this virus, and the potential for subsequent human cases of disease in Europe increased at this time, with particular UK discussion over the role of native mosquitoes as potential vectors. An entomological review [44] highlighted that *Culex pipiens* biotype *molestus* Forskal was the only native human-biting *Culex* species in the UK at that time, and that other non-*Culex* species that acquired blood meals from birds and humans may also be implicated as bridge vectors for WNV. This paper reported that *Cx. modestus* (Ficalbi) (a known vector elsewhere in Europe [45]) had been reported in southern England during the 1940s and it was not considered to have survived here. Since then, WNV outbreaks frequently occur in Europe, with 200–800 cases being reported each year [1], largely across Eastern and southern Europe, with the occasional outbreak being associated with horses in France.

#### 2.2.1. The Detection and Expansion of *Culex modestus* in the UK

In 2010, the HPA (now PHE) initiated trap-based native mosquito surveillance across a range of nature reserves, to better understand of our native mosquito fauna, their abundance, and their range. Since 2005, an additional four species of mosquito have been added to the UK list: *Ae. geminus* Peus, *Cx. modestus*, *Ae. albopictus*, and *Ae. nigrinus* (Eckstein) [42,46,47,48]. Most significant for WNV risk, was the finding of *Cx. modestus* in a trap in 2010 in North Kent. Survey work had previously been conducted by several research groups in the North Kent area, without any previous reports of *Cx. modestus*. One study in 2003 reported large numbers of the ornithophagic *Cx. torrentium* Martini on Sheppey [49], and given the mammal-lure used in the trap, it is possible that these were actually the first evidence of *Cx. modestus*, and they may have been overlooked.

**Figure 3:**
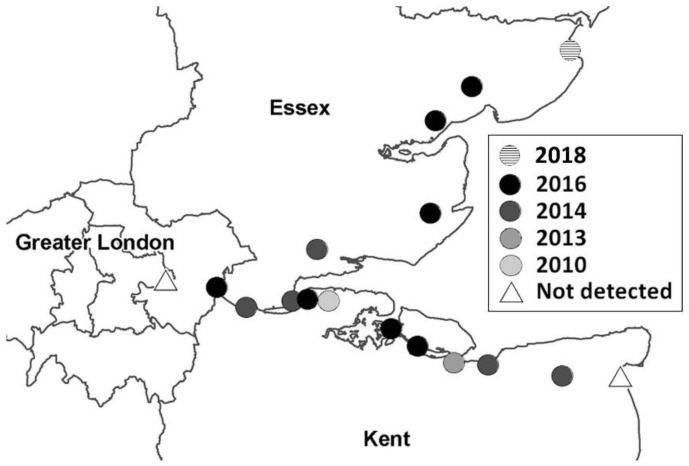
Distribution of *Culex modestus* mosquito in the Thames Estuary, up to July 2018.

Follow up surveys of *Cx. modestus* have been conducted by PHE [50,51,52,53] and colleagues at University of Greenwich and the distribution of this species is now known to extend along ditches across grazing marshes in North Kent between Swanscombe (near Dartford) and Stodmarsh (near Canterbury), with no evidence of presence south of the North Downs. In Essex, it was first reported near Basildon in 2014, and, as of July 2018, it occupies a range from Rainham in the west to Fingringhoe and Horsey in the north (Figure 3). A ring of traps surrounding this endemic area has so far found no evidence of the mosquito in the Norfolk Broads, Romney Marsh, Sandwich, or in East London.

*Culex modestus* may spread to similar habitat further up the Essex and Suffolk coasts, and field surveys to test this are ongoing. Its mode of spread is enigmatic; in fact, the apparent spread may be a reflection of the widening surveillance zone. However, it appears that movement of the mosquito from one suitable coastal habitat to another may be facilitated by birds, perhaps carrying eggs on their feet; however this remains a theory. Its reported establishment in Wallasea Island in 2016 and increased abundance in 2017 is in contrast to surveys there in 2011 [54] that reported an absence of the mosquito prior to the transformation of the island from an arable landscape to a newly created coastal wetland habitat. There are other individual records from the Cambridgeshire Fens and Poole harbour [50], but further sampling has never been able to confirm the local populations.

Recent field studies on *Culex modestus* in North Kent confirm both its ornithophagic habit [55] and that it is a prolific human biter [56]. Efforts to detect WNV in mosquitoes and birds by APHA and PHE [52,57,58] have found no evidence of virus transmission or exposure, although previous work [59] reported evidence that UK birds might have been exposed to the virus; although, no further evidence has been published since. Clinicians who observe patients with viral encephalitis and who are located in, or have visited, *Cx. modestus* infested areas are being asked to consider a WNV diagnosis and submit samples for testing at PHE. Further field studies to detect any exposure of horses to WNV in the infested region are encouraged.

#### 2.2.2. Environmental Change and British Mosquitoes

Although there is no current evidence of human WNV circulation in the UK [52,55], we should not be complacent. The UK has a large programme of wetland creation. This includes: (a) realigning coastlines for habitat creation and flood alleviation, (b) reverting arable land to wetland to expand the size of nature reserves for biodiversity, thus minimising habitat fragmentation in the face of climate change, (c) developing new urban wetlands to mitigate the effects of flooding, and (d) provide sustainable urban drainage and act as receptor sites for mitigating the loss of habitat for protected species. The impacts of all these strategies have been assessed with respect of UK mosquitoes [54,60,61,62]. Ensuring that wetlands are created and managed so as not to exacerbate future nuisance or disease risk should be a focus of all habitat management plans and disease risk assessments [62]. Developing guidelines for assessing mosquito risk is now a main theme of the Natural Environment Research Council (NERC) funded Wetland Life project.

Enhancing surveillance for mosquitoes and for WNV is a priority if we are to prepare for future outbreaks, and have the means and knowledge to minimise transmission and control problem mosquitoes. Future research that investigates the impact of biocidal treatments on native mosquitoes, insecticide resistance within those mosquitoes, and vector competence studies are required. Current evidence [63] suggest that UK mosquitoes, such as the saltmarsh mosquito *Aedes detritus* (Haliday), might be competent for WNV in the laboratory, however this does not mean that they will act as efficient field vectors, and further work on this is ongoing, as stated previously. The story of *Culex modestus* illustrates the need for ongoing surveillance of native UK mosquitoes, and the key role that is played in this by nature reserve wardens and environmental health officers who run a network of traps for PHE. Only with up to date data can we truly assess disease risk, and respond to new findings.

### 2.3. Other Mosquito-Borne Infections of Potential UK Concern

#### 2.3.1. Malaria Risk for the UK

Malaria was previously endemic to parts of the UK during the 19th century, with isolated outbreaks in the 20th century. Owing to the fact that there are six resident and potentially competent anopheline mosquito vector species, where the current climate is considered to be permissible for transmission, and that infected travellers return to the UK regularly, the possibility already exists for transmission locally [64]. Apart from the occasional cryptic case of malaria (associated with travelling), we continue not to see endemic malaria transmission in the UK. With climate change, theoretically a warmer summer would reduce the extrinsic incubation of the pathogen in mosquito and increase the local malaria risk, although there should be sufficient controls in place to prevent much onward transmission [39].

Modelling [65] investigated the impact of different climate change scenarios on the likelihood for *P. falciparum* transmission. Four of their five models suggested a low risk by 2100, even at extreme scenarios, with the fifth model predicting suitability in southern England for sustained transmission lasting more than one month by 2080. A model developed for *P. vivax* [66] predicted two months of climatic suitability in Great Britain and four months of climatic suitability in southeast England by 2030. So far, we are not seeing regular endemic malaria transmission across the Mediterranean Basin, although the recent outbreaks of vivax malaria in Greece [4] suggest that we need to remain vigilant. *Plasmodium falciparum* and *Plasmodium vivax* are not zoonotic, and the incidence of nuisance biting by UK anophelines remains low. Coupled with our ability to treat malaria cases (in contrast to certain arboviruses), the risk of local transmission also remains low. Studies that focus on anopheline biting habits (which for some appear to have changed [62]) and vector competence of urban anophelines (i.e., *Anopheles plumbeus* (Stephens) and *Plasmodium falciparum*) are worthy of further consideration. *Anopheles plumbeus* occurs widely in treeholes (and occasionally containers) in urban areas in the UK [62] and recent literature on its role as a potential malaria vector in Belgium should not be ignored [67].

#### 2.3.2. Other Arboviruses

A number of other mosquito-borne pathogens, including both arboviruses and filarial nematodes, could potentially be transmitted in the UK. An ecological and entomological assessment of a range of arboviruses concluded that mosquito species with the potential for transmission of Sindbis virus, Tahyna virus and Usutu virus occur in the UK [44,68]. Although none of these viruses is considered a major public health threat currently, the possibility for human cases justifies surveillance in both birds and humans to determine whether these viruses are circulating locally. It is possible, particularly in the case of Sindbis virus, that the ecology of transmission cycles in Scandinavia (where enzootic cycles are being amplified in Tetraonid birds and migratory thrushes) might be a limiting factor for transmission in the UK [68]. However, given that there are differing transmission cycles in Sweden as compared to Finland, then the potential for a novel transmission cycle in the UK still potentially exists. So far, there is no evidence for transmission of any of these arboviruses in the UK [27,69], and recent laboratory studies suggest that UK *Cx. pipiens* is not a competent vector of Usutu virus [70].

Rift Valley fever virus (RVFV) does not occur in Europe; and, although one of the primary vectors, *Aedes vexans* (Meigen) has been reported in the UK historically, until recently there had been no known viable population being described for 90 years. However, in 2017, populations were found in the Norwich area, causing nuisance biting [71]. Although this does not change the risk assessment (as RVFV remains absent from Europe), it does highlight that ongoing surveillance for native mosquitoes and monitoring of the situation elsewhere in Europe is required to inform ongoing disease risk assessments. Vector competence work testing whether native mosquitoes can transmit RVFV is also ongoing [72].

#### 2.3.3. Dirofilaria

*Dirofilaria* is transmitted by mosquitoes in large parts of the world, including the Mediterranean Basin, with *Dirofilaria immitis* the cause of heartworm in dogs, and both *D. immitis* and *D. repens* causing subcutaneous, ocular, and pulmonary dirofilariasis in humans [73]. There are several UK *Aedes* and *Anopheles* mosquitoes that theoretically could transmit *D. immitis* in the UK, with sufficient summer temperatures to support transmission [74]. Although it has not proven an issue so far for the UK, it should remain on the UK radar, as there has been an increase in transmission of this pathogen in Italy associated with locally established populations of imported *Ae. albopictus* [73].

## 3. Ticks & Tick-Borne Diseases

**Figure 4:**
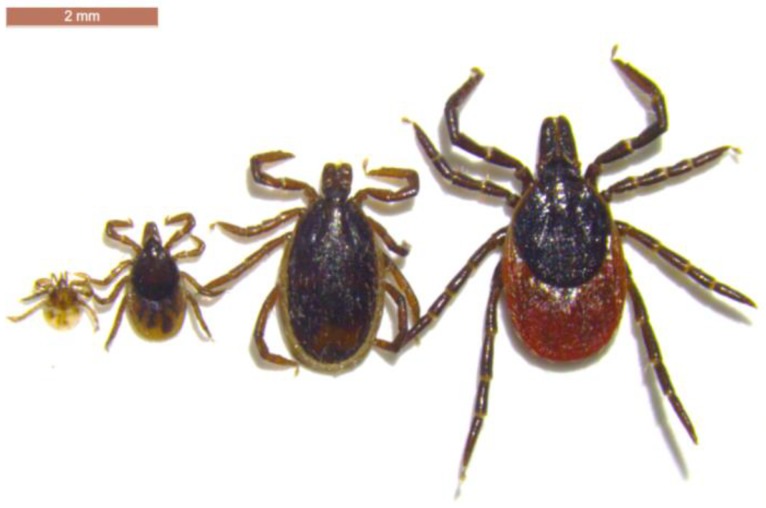
Three active life stages of *Ixodes ricinus* (from left: larva, nymph, adult (male, female)).

There are more than 20 species of native tick in the UK, with many being associated with particular wildlife species, such as seabirds, bats, tree-hole nesting birds, cliff-nesting birds, and small mammals [75,76]. There are a few species that do bite humans, and these also tend to be the most common species. The main tick species of public health concern is the sheep/deer tick, *Ixodes ricinus* L. (Figure 4), which is reported to be changing its distribution across Europe [77]. There are also reports of human biting from the hedgehog tick, *Ixodes hexagonus*, the red sheep tick *Haemaphysalis punctata* Canestrini & Fanzago, and very occasionally from the Ornate Cow tick *Dermacentor reticulatus* (Fabricius) [78,79,80,81]. Data on the distribution of our native and indeed non-native tick species have been recorded for over one hundred years [82]. For the last 14 years, a more formal surveillance system has been in place, organised by PHE, receiving tick records and samples from the public, general practitioners, veterinarians, academics, and wildlife charities. This dataset and resource has been crucial in enabling up-to-date maps to be created and changes monitored for our most common species, as well as identifying new foci for species previously considered local or rare. This data, at a local level, has been used to inform the public, local authorities, and environmental organisations, and at a national level to inform government disease risk assessments, HAIRS and ACDP. On an annual basis, the data that is collected are used to target enhanced surveillance and direct research. A summary of the more important findings from analysis of this dataset forms the basis for the rest of this section.

### 3.1. Ixodes ricinus and Lyme borreliosis

Lyme borreliosis is already a significant and growing public health concern for the UK (as well as elsewhere in Europe), with the transmission of *Borrelia burgdorferi s.l.* between wildlife and to humans facilitated by the involvement of its primary UK vector, *Ixodes ricinus* and several cycles involving small mammals (such as *Apodemus sylvaticus*, *Apodemus flavicollis*, and *Myodes glareolus*), squirrels, woodland birds, and gamebirds and their involvement with the circulation of different genospecies of *Borrelia burgdorferi s.l.*: including *B. garinii*, *B. afzelii*, *B. burgdorferi s.s.*, and *B. valaisiana* the dominant genospecies across the UK [83,84,85,86,87,88,89]. There are, however, also lesser (currently) infectious disease concerns associated with this and other less prevalent tick species, including those being imported into the UK via wildlife (migratory birds) or companion animals. Consequently, this section focuses on *I. ricinus* and Lyme borreliosis with later sections considering other potential tick borne diseases that could be vectored by this and other tick species in the UK.

#### 3.1.1. Expansion of *Ixodes ricinus* Ticks

Distribution maps for *I. ricinus* have been published in recent years: in 2005 [90], 2011 [78], and 2018 [90]. In the most recent publication, data was published on more than 14,000 ticks submitted to the scheme between 2010–2016, recording 11 native and 17 non-native species. The most common tick recorded (60%) was *I. ricinus*. In the UK this species is the primary vector of Lyme borreliosis, caused by the bacterium *Borrelia burgdorferi s.l.*, being responsible for >1000 laboratory confirmed cases each year in England and Wales [91], and possibly several thousand non-confirmed cases.

**Figure 5:**
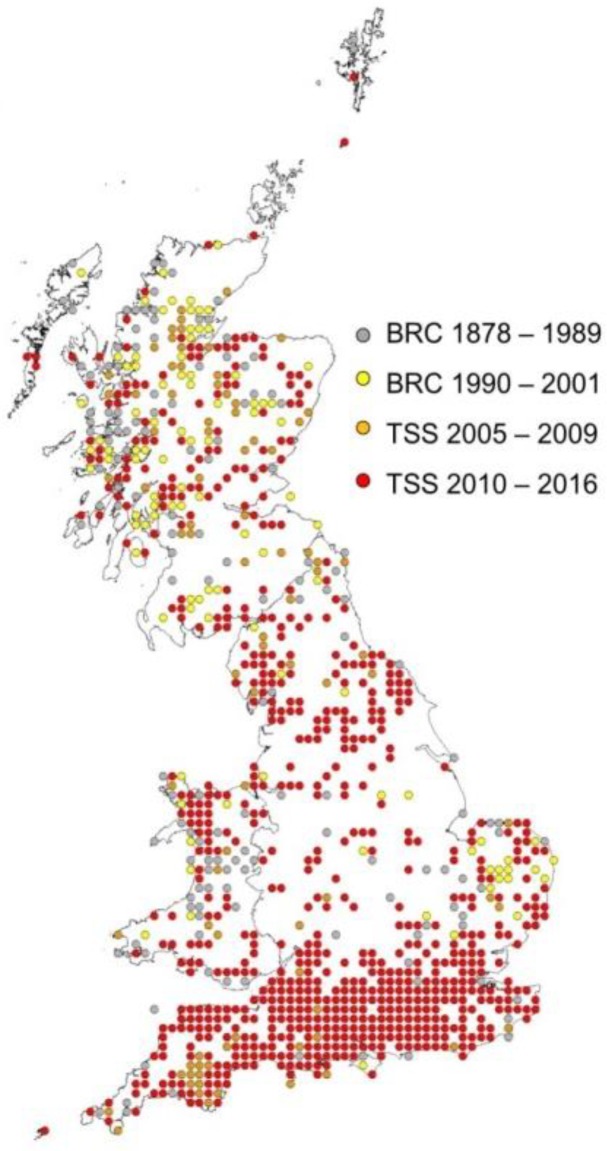
Distribution of *Ixodes ricinus* ticks in Great Britain (after [79]).

An assessment of the change in distribution of *I. ricinus* has been published [79], comparing the number of 10 km^2^ squares containing a record of *I. ricinus* in 2016 with the reported distribution in 2010 and 2005 (Figure 5). Although an admittedly crude assessment, it does provide the first semi-quantitative information on changes in distribution of this tick. In England in 2010, there were records of *I. ricinus* in just 20% of 10km grid squares [78], but this had increased to 42% by 2017, suggesting that tick records are now being reported from a wider geographical range across England. This increased reporting of tick records may be due to greater awareness and coverage of the Tick Surveillance Scheme (TSS), but anecdotal information through the scheme also suggests that changes in distribution are actually occurring. Breaking this data down by PHE region, changes in the distribution of tick record coverage have increased from 39% to 65% in southwest England and from 26% to 67% in southeast England. Similar orders of magnitude of change appear to have occurred elsewhere; with Wales increasing from 25% to 37% of grid squares reporting the tick over the same time period. Areas of England with lower proportions of grid squares reporting *I. ricinus* remain the Midlands, with 14% of the West Midlands and 13% of the East Midlands having 10 km grid squares with records of *I. ricinus*. Other areas of the country, such as the southern Lake District, Cheviots, North Yorkshire Moors, and Thetford Forest however also remain important areas of *I. ricinus* distribution. Of course this data does not necessarily reflect tick abundance, but it does highlight possible changes in distribution due to the new records in many new geographic areas, which facilitates increased local awareness and the potential targeting of a response.

#### 3.1.2. Urban Tick Issues

These potential changes in the tick’s distribution and the increasing interest being shown by the public are reflected in the significant number of that were enquiries made to PHE through the TSS. There is, for example, an increasing trend towards issues being reported in parks, gardens and urban greenspace. A preliminary assessment of the suitability of urban greenspace for ticks in the UK has therefore been conducted, and in Salisbury [92], urban woodlands and woodland edge were identified as key habitats, with mean prevalence rates of *Borrelia*-infected *I. ricinus* ticks at 18%, and with the highest prevalence for a single location being reported as 30% of ticks infected.

PHE is continuing similar studies of ticks and their *Borrelia* carriage rates in other urban greenspaces to establish the more general UK picture; in this instance, including Bath, Bristol, Southampton, and London, funded by the Health Protection Research Unit on Environment and Health, in collaboration with the University of Exeter. One of the challenges for local authorities is how to manage urban greenspace to minimise exposure of the public to tick bites, and how to raise public awareness of ticks without causing undue alarm that could hamper other advances in public health, such as the realisation of the health benefits of the use of greenspaces by the public [93]. A toolkit for local authorities and environmental organisations to assist with messaging around public awareness of ticks and Lyme borreliosis has recently been produced by PHE (https://www.gov.uk/government/publications/tick-bite-risks-and-prevention-of-lyme-disease).

Local authorities are now being encouraged to use this toolkit to develop local tick awareness materials for local campaigns. Gathering further evidence concerning the factors affecting the distribution of *I. ricinus* in urban greenspace is consequently crucial. For example, it is expected that well connected woodlands and woodland edges might harbour ticks, but it is less clear whether this might apply in large urban parks or managed meadows with long grass. It is also possible that the management of these spaces for biodiversity could be inadvertently creating habitats for ticks, as they are designed to provide habitats for their mammal and avian hosts [94]. Novel strategies of habitat management could therefore be developed to minimise tick exposure, whilst maximising biodiversity, as has been suggested for woodland rides [95]. Further, if wildlife corridors are encouraging the incursion of key tick hosts, such as deer, into urban areas, studies are needed to determine if we need to, and how we can, best manage deer populations or their movements [96] to help minimise tick bite and Lyme disease risk.

We know from work in urban areas in Salisbury that prevalence rates for *Borrelia*-infected ticks can be high in comparison to studies that were undertaken in rural areas [9,97]. We need to know whether this is reflected across other urban areas and whether it could be due to a lack of a dilution effect on infection rates in the absence of large mammals, in that livestock and deer tend to be less abundant in urban areas. The basis for this hypothesis is that sheep, cattle, and deer, whilst being important tick hosts, play little to no role in infecting ticks with *Borrelia*. Studies elsewhere have shown that *Borrelia* prevalence rates can be lower outside deer exclosures (where deer are excluded) when compared to inside exclosures where ticks acquire their bloodmeal from infected small mammals and birds [98]. Therefore, in an urban setting, is it possible that *Borrelia* prevalence rates may be higher than in the wider countryside, albeit that ticks are at lower abundance, or are we likely to see wide variations in prevalence rates in different urban settings? Ongoing work is being undertaken to better understand these factors and their potential impacts on public health, given that the public are probably more frequently engaged in activities in urban greenspaces than in the wider countryside.

#### 3.1.3. *Borrelia* Prevalence in Ticks & Impacts of Environmental Change & Wildlife Management

**Figure 6:**
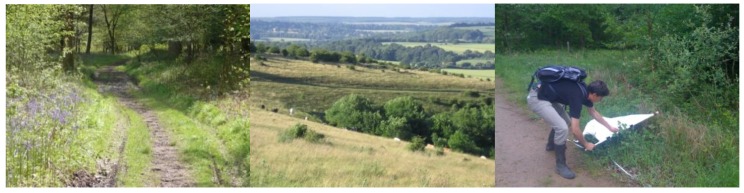
Main habitats for *Ixodes ricinus* ticks in woodland, grazed grassland. Ticks are collected by blanket dragging vegetation.

Ongoing work by PHE is aiming to better understand the prevalence of *Borrelia* infection in ticks (as well as *Borrelia* genospecies dominance by genetic sequencing) across landscapes, with data now being available over a five-year period for an area of rural South Wiltshire (Figure 6). Early unpublished results suggest mean prevalence rates of *Borrelia*-infection in *I. ricinus* of up to 13% that varies between years, with again up to 30% prevalence being reported in some localities. In the countryside, there are also other potential issues to consider, such as how increasing numbers of deer and game bird releases may be facilitating the spread of ticks, as well as impacting on *Borrelia* transmission cycles. Previous studies on ticks and *Borrelia* associated with pheasant have highlighted that these game birds can be important hosts for nymphal ticks, and they may be involved with amplifying *Borrelia garinii* cycles [88]. However the timing of their release from August onwards does not coincide with the main *I. ricinus* activity period, and there are suggestions that pheasant may play an important role in mopping up nymphs from vegetation and reducing exposure of people and other hosts to tick bites. Further studies are required to better understand the role of game bird releases and increasing deer numbers on the eco-epidemiology of *Borrelia*.

### 3.2. Ixodes ricinus and Other Known or Potential Pathogens

#### 3.2.1. *Anaplasma*, *Borrelia miyamotoi* & *Rickettsia*

We should also remain mindful that *Borrelia burgdorferi* is not the only known or potential pathogen transmitted by *Ixodes ricinus*. Pathogens, such as *Anaplasma phagocytophilum*, *Babesia* sp., and louping ill virus have long been known to be present in *I. ricinus* populations in the UK [99,100]. There has been very little studied on the ecology of *A. phagocytophilum* in the UK. Previous papers have reported prevalence rates of 6–7% in field voles [101], and 2% in *Ixodes ricinus* ticks [9]. It is the causative agent of human granulocytic anaplasmosis in other parts of the world, and although it is widely reported in animals [102], human cases in Europe are not frequent, although they may be underestimated due to their nonspecific flu-like symptoms. More recently, evidence of *Rickettsia helvetica* and *Borrelia miyamotoi* infection in *I. ricinus* in the UK has been reported [9,10,103]. Although they are considered to be pathogens elsewhere, it is unclear whether they cause clinical disease in humans in the UK [39]. Further work is ongoing to determine any pathogenicity for these agents and ensure the diagnosis of potential cases that might otherwise go undetected.

#### 3.2.2. Tick-Borne Encephalitis and Congo-Crimean Haemorrhagic Fever Viruses

Studies are also ongoing with respect to the potential for tick-borne encephalitis virus (TBEV) in the UK, with laboratory testing for evidence of the virus in native questing ticks, ticks on migratory birds, and from serological studies of deer blood. These investigations are particularly pertinent given the recent evidence of TBEV-positive deer sera and TBEV-positive ticks in the Netherlands [104]. Non-native ticks are also being imported into the UK on migratory birds, including *Hyalomma marginatum* Koch, and this remains the most likely route by which novel arboviruses, such as Crimean Congo haemorrhagic fever virus (CCHFV), may be imported [105,106]. These two tick-borne viruses are emerging in new geographic areas in Europe, and ongoing surveillance for the virus and their vectors in the UK remains a priority.

### 3.3. Dermacentor reticulatus and Associated Infections

The TSS has detected potential changes in the distribution of two other, non-*Ixodes* tick species with disease vector potential: *Dermacentor reticulatus* and *Haemaphysalis punctata*. These two species exhibit different seasonalities to *I. ricinus*, and they are found in differing habitats, making accurate assessments of distribution and vector status critical to any public and veterinary health communication. The Ornate Cow tick, *D. reticulatus* has historically been reported in a number of coastal sites in west Wales and north and south Devon. It is primarily associated with sand dune habitat, but also coastal grassland. A recent paper reviewing the historical data on *D. reticulatus* confirmed that there are established populations in three main areas of England and Wales: along the West Wales coast from Harlech to Borth, on the North and South Devon coasts centred around the sand dunes near Bideford, and coastal grassland at Bolt Tail, with a third focus in Essex, in both coastal grassland and urban parks [80]. The first report of *D. reticulatus* being found in Essex was published in 2009 on Potton Island associated with the movement of Welsh sheep to an area with horses [107]. Since then, the tick appears to have spread, possibly on both horses and livestock to other areas along the Essex coast, some of them nature reserves, but also to urban greenspace in Harlow [108,109].

These findings present a few concerns. Movement of animals may be responsible for moving these ticks between sites, and consideration ought to be given to ensuring that livestock and horses moved from endemic areas for conservation purposes or recreation are not inadvertently transporting ticks to new areas where the tick can establish locally [80]. This could be achieved by modifying the timing of moving flocks and herds to a time of low tick activity (peak activity of the adult ticks occurs in March/April) or targeted acaricidal treatment. This issue is further complicated by the emergence of canine babesiosis in Essex, associated with bites from *Babesia canis* infected *D. reticulatus* on dogs. Several canine cases, including a fatality, have been reported in the Harlow area, in which a high prevalence of infected *D. reticulatus* ticks (85%) was reported [108,109,110]. It is likely that without controls, this tick will spread to many public areas and coastal grasslands within Essex and beyond. If they continue to be implicated in the transmission of *Babesia canis*, this presents a potentially very serious veterinary health issue, although as this is not zoonotic it is not a public health threat.

The detection of *Rickettsia raoultii* in the UK populations of *D. reticulatus* [10,103] and the importation of *R. slovaca* infected *D. marginatus* Sulzer [111] is a potential public health threat. These rickettsiae are responsible for a syndrome in humans that is characterized by scalp eschars and neck lymphadenopathy (SENLAT), previously known as tick-borne lymphadenopathy (TIBOLA) or *Dermacentor*-borne necrosis erythema and lymphadenopathy (DEBONEL) [112,113]. Managing tick populations now and minimising their spread could prevent further dissemination and minimise any present or future disease risk.

### 3.4. Haemaphysalis punctata and Potential Disease Issues

There has been much interest recently in North America over the detection, spread, and health risks that are associated with *Haemaphysalis longicornis* Neumann [114]. This species has not been detected in the UK, however another species of *Haemaphysalis* has been identified as a potential emerging health issue. The red sheep tick, *Haemaphysalis punctata*, has been reported to occur in England for over the last 100 years. However, recent evidence from the TSS shows a greater frequency of submissions in the last few years and an increased number of reports of human biting [79]. Recent evidence [81] suggests an apparent spread of *H. punctata* in the eastern part of the South Downs in East Sussex. Although there are historic records in this area, an increase in reporting around the Lewes—Eastbourne area suggest that these ticks are more abundant than they were and they are causing increased human biting. Anecdotal evidence from farmers is being reported that ewes with heavy tick burdens are giving birth prematurely, with significantly higher numbers of abortions, particularly in naïve sheep that are new to the area. Whether *H. punctata* is involved remains uncertain, however the increased number of records coming into the TSS, including cases of human biting, and the increased number of confirmed established populations identified through field surveys suggest that the status of this tick may be changing [81]. Based on the current distribution of the tick and its observed habitat preference, with tick density of *H. punctata* highest in grazed grasslands, it would seem likely that *H. punctata* may spread to other sheep-grazed grasslands across East Sussex and perhaps beyond. This apparent movement might be being facilitated by the movement of infested sheep and/or birds. Work to better understand the role of sheep and birds as tick and disease vectors is now crucial, particularly as humans presenting with *H. punctata* bites are also being reported to have Lyme borreliosis.

### 3.5. Imported Ticks: Rhipicephalus sanguineus s.l.

Following the harmonisation of pet travel regulations in 2012, tick controls on pets travelling to or from Europe were no longer required. Since 2012, there has been a trend for an increasing number of imported and travelling dogs infested with the brown dog tick, *Rhipicephalus sanguineus s.l.* [115,116]. Numerous papers have reported imported ticks, sometimes with high numbers of both male and female *R. sanguineus s.l.* Latreille on the same dog. On some occasions, importations of these non-native ticks have led to house infestations [117]. These Mediterranean ticks are not thought to survive outdoors in the UK, but they can complete their life cycle indoors, laying many thousands of eggs, with ticks being able to survive long periods without a blood meal, infesting furniture, and living behind skirting boards and wallpaper. We strongly recommend re-instating tick controls on travelling and imported pets to minimise imports and house infestations and mitigate any associated disease risks, as this tick is a known vector of *Rickettsia conorii*, the causative agent of Mediterranean spotted fever. 

**Figure 7:**
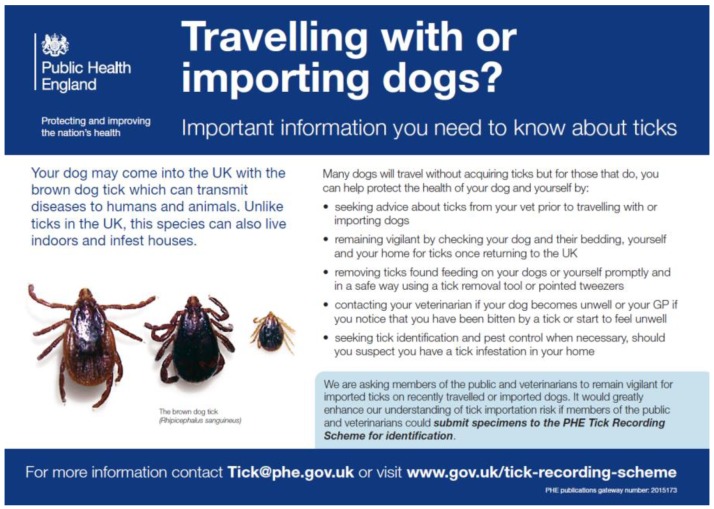
Raising awareness of risks associated with native, and, in this case, non-native ticks.

Imported ticks on both animals and humans remain a potential risk to the UK. A few examples of the ticks imported into the UK, which are associated with potential public and veterinary health risks include: imported *Hyalomma marginatum* on horses from Portugal [118], *Hyalomma lusitanicum* Koch on a travelling dog from Portugal [119], *Dermacentor marginatus* and associated *Rickettsia slovaca* imported possibly in luggage from central Europe [111], and the tick-borne paralysis vector *Ixodes holocyclus* Neumann from Australia [120], although technically not a disease vector. A recent summary [116] of the ticks imported into the UK highlights the increasing incidence and potential risk associated with the various tick species that are arriving. Increased awareness amongst pet owner and pet charities is needed (Figure 7), particularly in the absence of legislation changes that might reinstate tick treatments on travelling pets.

## 4. Conclusions 

Our ability to assess vector-borne disease risks ahead of time, especially in the face of new findings or the initiation of an outbreak, is contingent on: robust systems of monitoring and surveillance of vectors, research on the ecology of those vectors and their associated pathogens, models that consider the impact of weather and climatic change, laboratory testing of vectors for pathogens, and studies of their vector competence. Capacity and resource in these areas is essential to provide informed rapid assessments concerning public and veterinary health risk and to proportionately inform potential control and mitigation strategies. The more that we conduct surveillance and research in this area the better we understand the complexity of the eco-epidemiology of vector-borne disease, and the contingent issues that could arise. Being better prepared, the better chance we have of staying ahead of the curve. It is crucial that we continue to maintain and build UK medical entomology capability that is engaged across government, academia, and internationally to address key emerging issues, many of which may challenge the current norm. Then, we will be in a better position to rapidly respond to emerging issues, ensuring that we are able to manage the changing risk from these complex infectious diseases, and their arthropod vectors.

This paper summarises the key issues arising from the latest surveillance data and related research being undertaken in the UK and abroad that could potentially help to inform discussion and action concerning current and future vector-borne disease risk to the UK. It supports the need for continued vector surveillance systems to monitor our native arthropod vector fauna, as well as the need to expand surveillance for invasive mosquito and tick species. It illustrates the importance of building and maintaining local surveillance capacity as well as the necessary networks across Europe and internationally, which is sufficient to ensure accurate and timely disease risk assessment to help mitigate the risks from the UK’s changing emerging infectious disease risks, particularly in the light of climate change and increasing global connectivity.
